# The diagnostic odyssey of autism: a cross-sectional study of 3 age cohorts of children from the 2016–2018 National Survey of Children’s Health

**DOI:** 10.1186/s13034-021-00409-y

**Published:** 2021-10-10

**Authors:** Allison Hanley, Quynh C. Nguyen, Deborah Golant Badawi, Jie Chen, Tianzhou Ma, Natalie Slopen

**Affiliations:** 1 Department of Epidemiology and Biostatistics, University of Maryland School of Public Health, College Park, MD USA; 2 Division of Developmental-Behavioral Pediatrics, University of Maryland School of Medicine, Baltimore, MD USA; 3 Department of Health Policy and Management, University of Maryland, College Park, MD USA; 4 Department of Social and Behavioral Sciences, Harvard T. H. Chan School of Public Health, Boston, MA 02115 USA

**Keywords:** Autism spectrum disorder, Diagnosis, Early intervention, Early identification, Diagnostic odyssey

## Abstract

**Background:**

Autism prevalence has increased rapidly in recent years, however, nationally representative estimates on the ages of first identification and intervention are out of date. Objectives: (1) To estimate the ages at which children with autism receive their first diagnosis, intervention plan, and developmental services; and (2) To evaluate differences in ages at events by birth cohort and sociodemographic characteristics.

**Methods:**

Using cross-sectional data from the 2016–2018 National Survey of Children’s Health (NSCH), we examined associations via linear regression among a sample of 2303 children aged 2–17 years old, who had ever been diagnosed with autism and either (1) ever had a plan for special education or early intervention, or (2) ever received special services to meet developmental needs. Exposures included age cohort, child, household and healthcare provider characteristics.

**Results:**

Most children in the study sample (n = 2303) were over age 6 years, male, of non-Hispanic white race/ethnicity and had mild/moderate autism. Mean ages (years) at first diagnosis was 4.56 (SE = 0.13); first plan was 4.43 (SE = 0.11); and first services was 4.10 (SE = 0.11). After adjustment for exposures and survey year, the middle childhood cohort was 18 months older at first intervention (β = 1.49, 95% CI, 1.18–1.81), and adolescents were 38 months older at first diagnosis (β = 3.16, 95% CI, 2.72–3.60) compared to those in early childhood. Younger ages at events were observed among: Hispanic/Latinx as compared to white children, those with moderate or severe symptoms as compared to mild symptoms, and children who received their diagnosis from a specialist as compared to psychologists or psychiatrists.

**Conclusions:**

Children with autism receive their first diagnosis, intervention plans and developmental services at younger ages than they had in the past. Future research is needed to identify the mechanisms for these improvements in early identification and intervention to accelerate additional progress.

**Supplementary Information:**

The online version contains supplementary material available at 10.1186/s13034-021-00409-y.

## Background

Autism spectrum disorder (ASD) is a neurodevelopmental disorder characterized by difficulties with social communication and restricted interests or repetitive behaviors [1]. Since 2011, the prevalence of autism has risen over 122%, and recent epidemiologic studies estimate the prevalence among children aged 3–17 in the US is 1.74% [2]. Between 2009–2017 the overall prevalence of ASD was significantly lower among non-Hispanic Black children (1.54%) and Hispanic children (1.34%) when compared to non-Hispanic white children (1.95%) [2]. Earlier intervention is associated with better long-term outcomes in domains such as cognition, daily living skills, severity of symptoms, and peer relationships [3].

Prior to beginning early intervention, there must first be a recognition that the child would benefit from developmental supports. The process of identifying concerns about a child’s behavior or development, obtaining a professional evaluation, and accessing developmental intervention services can be lengthy, confusing, and frustrating for families. This journey from concern to diagnosis to intervention is arduous enough that it has been dubbed “the diagnostic odyssey”[4].

The most recent nationally representative data on the events of the diagnostic odyssey of autism comes from the 2011 Survey of Pathways to Diagnosis and Treatment (hereafter referred to as the Pathways Survey). Children in the Pathways Survey were aged 6–11 at the time of the survey (i.e., born between 2000–2005). Children with autism in the Pathways Survey (n = 1420) were estimated to be 3.90 years old (SE = 0.11) when they first began receiving specific services to meet their developmental needs [5], and were 5.2 years (95% CI, [4.9–5.5] when their parents were first told their child had ASD [6].

Research on the factors associated with age at events in the diagnostic odyssey has produced mixed results. Studies using regional samples have found racial/ethnic disparities in the age of diagnosis [7–9]. The majority of studies in the US found that non-Hispanic white children were diagnosed earlier than non-Hispanic Black children [7, 8, 10]; Hispanic children [10–12] and children of other racial/ethnic groups [13]. One study found that non-Hispanic white children were diagnosed later than Hispanic children [12]. However, earlier studies using nationally representative data did not observe these racial/ethnic differences [14]. Studies that have examined socioeconomic status (SES), the presence of an older sibling, and the setting in which a child receives a diagnosis (e.g., healthcare vs educational) have also led to mixed results [8, 11, 13, 15–17].

Autism prevalence has increased in the past decade [2] alongside increases in rates of developmental screening in early childhood [18]. However, it is unclear if children are identified or begin intervention earlier. The objectives of this cross-sectional study are (1) to estimate the ages at which children with autism receive their first ASD diagnosis, intervention plan and developmental services using a nationally representative sample of children from a wide birth cohort, and (2) examine associations between ages and sociodemographic and healthcare characteristics. Updated estimates of ages at which children experience events along the diagnostic odyssey using a recent and nationally representative sample will provide insight on progress and social and demographic patterning of ages at diagnosis, plan, and services for children with ASD.

## Methods

### Data source and study population

Data for this study came from the 2016–2018 National Survey of Children’s Health (NSCH). The NSCH is a nationally representative parent-completed survey of children in the US conducted since 2003 by the Census Bureau under the direction of the Health Research Services Administration. Questions pertain to the health and well-being of non-institutionalized children under the age of 18. The survey is designed to oversample children aged 0–5 and children with special healthcare needs [19]. In 2016 the NSCH was redesigned to replace telephone administration with mail and web-based questionnaires and to collect data annually rather than every four years. Address data from the Census Bureau was used to determine which addresses in the US were more likely to have children aged 0–17, and households were then randomly selected for screening. Those eligible households with children had one child randomly selected to whom the survey questions pertain [19]. Parents provided informed consent to participate and provide responses regarding their children's health and well-being. As data are publicly available and anonymized, the Institutional Review Board at the University of Maryland, College Park determined the study to be exempt from classification as human subjects research.

Participants had the option to complete the NSCH in English or Spanish, and telephone-based assistance was available for completing the survey in other languages. Surveys were completed between the summer and winter of 2016, 2017 and 2018 (N = 102,341). For households with a confirmed child the completion rate was 69.7% in 2016, 70.9% in 2017, and 78.0% in 2018 [20–22]. The overall response rate for all households was 40.7, 37.4 and 43.1%, respectively. All analyses were weighted to account for the unequal probability of selection and differential nonresponse, and therefore the data represent all non-institutionalized children under age 18 who are housed in the US.

Autism can be reliably diagnosed as early as 2 years of age [23] therefore, we set the age range for inclusion to 2–17 years old at the time of the survey (see Additional file 1: Appendix S1. Figure S1; N = 94,303), and required that parents had affirmed that a doctor or other healthcare provider had [ever] told them that their child had autism, autism spectrum disorder, Asperger’s disorder or pervasive developmental delay (hereafter collectively referred to as an *ASD diagnosis*; N = 2688). In addition to having been diagnosed with ASD, we required that parents reported that their child had experienced at least one of the two following events: (1) had [ever] had a special education or early intervention plan (hereafter referred to as an *intervention/education plan*; N = 2133) or had [ever] received special services to meet his or her developmental needs such as speech, occupational, or behavioral therapy (hereafter collectively referred to as *developmental services*; N = 2164). A total of 2303 children in the 2016–2018 NSCH had either an intervention/education plan or had received developmental services in addition to an ASD diagnosis.

### Outcomes

#### First ASD diagnosis

Parents are asked to report how old the child was (in years) when a doctor or other health care provider first told them that the child had an ASD.

#### First intervention/education plan

Parents are asked to provide the child’s age at the time of the first plan. Reported ages are provided by the NSCH in years for children who were older than age two at the time of the first intervention/education plan, and in years and months for children younger than 24 months at the time of first intervention/education plan. Ages were converted to months, then to decimal-year before mean age was calculated.

#### First developmental services

Parents were asked to provide the age their child was when they began receiving these developmental services (in years and months). Reported ages are provided by the NSCH in years for children who were older than age three at the time they first began receiving developmental services, and in years and months for children younger than 36 months at the time of first developmental services. Ages were converted to months, then to decimal-year before mean age was calculated.

### Sociodemographic and healthcare characteristics

Based upon previous literature [17] we selected characteristics related to the child, the household and the diagnostic experience to examine in relation to the age outcomes. Child characteristics include age cohort, sex, race/ethnicity, and parent-rated severity of ASD. Age cohort was determined by the selected child’s age at the time of the survey [24]. The Early Childhood cohort includes children born between 2013–2016 (e.g., 2–5 years old), the Middle Childhood cohort includes children born between 2007–2012 (e.g., 6–11 years old), and the Adolescent cohort includes children born between 2001–2006 (e.g., 12–17 years old). Household characteristics include the highest household education level, ratio of household income to the federal poverty level (hereafter referred to as *% of FPL*), family structure (i.e., the number of adult caregivers in the home) and the presence of an older sibling. For categories used for each covariate, see Table 1. Parents also selected the type of doctor or healthcare provider that first told them their child had an ASD from a list of six options.

**Table 1:** Descriptive characteristics of pooled sample, National Survey of Children’s Health, 2016—2018 (n = 2303)

	Total (N = 2303) or %	Total weighted % (95% CI)	Early childhood N (%)	Middle childhood N (%)	Adolescence N (%)	*p*-value^a^
Total	2303	2.43% (2.43, 2.44)	293 (13.8%)	840 (45.6%)	1170 (40.5%)	
Weighted population size, No	1,598,915		221,409	729,767	647,739	
Sex	n = 2303					
Male	1855	78.4%	240 (77.5%)	676 (77.2%)	939 (80.2%)	0.79
Female	448	21.6%	53 (22.5%)	164 (22.8%)	231 (19.9%)	
Race/Ethnicity	n = 2303					
Non-hispanic white	1584	45.7%	175 (39.9%)	543 (37.0%)	866 (57.5%)	** < 0.001**
Non-hispanic black	160	14.9%	29 (20.5%)	57 (13.9%)	74 (14.2%)	
Hispanic/Latinx	277	31.5%	48 (31.5%)	116 (39.4%)	113 (22.5%)	
Other or multiracial	282	7.9%	41 (8.2%)	124 (9.7%)	117 (5.8%)	
Severity of ASD	n = 2303					
Mild	1036	39.2%	109 (47.1%)	378 (36.3%)	549 (39.7%)	0.19
Moderate	858	42.4%	124 (33.0%)	326 (46.5%)	408 (41.1%)	
Severe	237	9.8%	39 (14.0%)	75 (7.6%)	123 (10.8%)	
Missing	172	8.6%	21 (5.9%)	61 (9.6%)	90 (8.4%)	
Parental education	n = 2298					
High school graduate (or less)	387	34.6%	52 (33.0%)	154 (41.4%)	181 (27.2%)	0.25
AA or Some college	595	23.5%	65 (25.0%)	218 (19.9%)	312 (27.2%)	
Bachelor’s degree (or higher)	1316	41.8%	176 (41.0%)	467 (38.7%)	673 (45.7%)	
Family income, % of FPL	n = 2303					
< 100%	321	26.5%	46 (29.0%)	131 (27.0%)	145 (25.6%)	0.47
100–199%	451	26.8%	57 (27.5%)	182 (30.8%)	212 (22.7%)	
200–399%	711	24.2%	99 (24.2%)	242 (21.3%)	370 (25.4%)	
> 400%	820	22.5%	92 (19.3%)	285 (20.9%)	443 (26.3%)	
Family structure	n = 2303					
Two-parent household	1684	69.8%	225 (67.7%)	615 (73.0%)	844 (67.0%)	0.42
Other household/missing	619	30.2%	68 (32.3%)	225 (27.0%)	326 (33.1%)	
Presence of older sibling	n = 2303					
Yes	563	36.2%	118 (48.5%)	285 (48.8%)	160 (17.8%)	** < 0.001**
No	1740	63.8%	175 (51.5%)	555 (51.1%)	1010 (82.3%)	
Type of healthcare provider to diagnose	n = 2303					
Specialist	822	32.6%	128 (40.0%)	291 (30.6%)	403 (32.3%)	0.26
Primary care physician	281	12.6%	38 (16.3%)	108 (12.5%)	135 (11.3%)	
School psychologist	257	14.3%	27 (10.8%)	99 (17.5%)	131 (11.9%)	
Non-school psychologist	378	13.6%	39 (15.5%)	149 (12.9%)	190 (13.7%)	
Psychiatrist	275	9.7%	20 (4.4%)	94 (8.7%)	161 (12.7%)	
Other, unknown or missing	290	17.3%	41 (12.9%)	99 (17.9%)	150 (18.1%)	

^a^Bolded *p*-values denote statistical significance at *p* < 0.05. *ASD* autism spectrum disorder. *FPL* % of federal poverty level

All analyses were weighted to account for the complex survey design

### Missing data

The NSCH uses multiple imputation methods to impute the missing values of % of FPL and hot deck imputation to impute the missing values of the selected child’s sex, race, and ethnicity [19]. Data on % of FPL were missing for 18.6% of all respondents in 2016, 16% in 2017 and 15.3% in 2018 [20–22]. To retain individuals with missing data values on covariates of family structure (N = 14, 0.61%) and the type of provider to diagnose ASD (N = 49, 2.1%), we categorized these individuals as Other/Don’t Know. A separate category was created for the missing values of autism severity variable (N = 172, 7.5%).

### Statistical analysis

Differences between the three age cohorts on the distributions of covariates were evaluated via chi-square tests of independence. To examine differences in ages at events for the three outcomes by social and demographic characteristics, we conducted three linear regression models: bivariate models, models adjusted for age cohort, and the full model including all covariates and the survey year. The results for the bivariate models and models adjusted only for age cohort are presented in Additional file 1: Appendix S2. Table S1.

All analyses accounted for the complex survey design of the NSCH in the estimation of variance by incorporating standardized weights for the stratum, primary sampling unit and subpopulation via the survey package in R version 4.0 [25]. Statistical significance was assessed at the level of α = 0.05 and 95% for confidence intervals.

## Results

The weighted sample represents roughly 1.6 million children in the US, which is 2.43% of all children aged 2–17 in the survey. Most children in our analysis were male (78.4%) and aged 6 years or older, with only 13.8% in the Early Childhood cohort (i.e., born between 2013–2015, 2–5 years of age at the time of the survey). See Table 1 for complete descriptive characteristics of the sample overall and stratified by age cohort.

Table 2 estimates the mean ages (in years) for each of the three events. Based on the estimated mean ages for the total sample the events occurred in the following order: first developmental services (*Mean (M)* = 4.10, Standard Error (SE) = 0.10) followed by first intervention/education plan (*M* = 4.43, SE = 0.11), then finally first ASD diagnosis (*M* = 4.56, SE = 0.13). As shown, the order of events changes when the sample is stratified by cohort. The mean ages at which each cohort experienced each event decreased from the oldest to the youngest cohorts for all three events.

**Table 2:** Mean ages at events, National Survey of Children’s Health, 2016—2018

	N	Weighted N	Weighted %	Age years (SE)	*p*-value^a^
First developmental services					
Full sample	2164	1,502,230	100%	4.10 (0.10)	
Early childhood	284	219,126	14.6%	2.48 (0.08)	Ref
Middle childhood	800	683,385	45.5%	3.98 (0.14)	** < 0.001**
Adolescence	1080	599,719	40.0%	4.81 (0.17)	** < 0.001**
First intervention/education plan					
Full sample	2133	1,467,511	100%	4.43 (0.11)	
Early childhood	260	202,267	13.8%	2.48 (0.09)	Ref
Middle childhood	779	666,613	45.4%	4.39 (0.18)	** < 0.001**
Adolescence	1094	598,631	40.8%	5.14 (0.15)	** < 0.001**
First autism diagnosis					
Full sample	2303	1,598,915	100%	4.56 (0.13)	
Early childhood	293	221,409	13.8%	2.38 (0.08)	Ref
Middle childhood	840	729,767	45.64%	4.26 (0.17)	** < 0.001**
Adolescence	1170	647,739	40.5%	5.64 (0.22)	** < 0.001**

^a^Bolded *p*-values denote statistical significance at *p* < 0.05. All analyses were weighted to account for the complex survey design

Table 3 displays the results of the multiple linear regression models used to estimate the adjusted relationship between the sociodemographic and healthcare covariates and each of the three events.

**Table 3:** Adjusted linear regression associations for ages at events, National Survey of Children’s Health, 2016—2018

	First developmental services (n = 2164)	First intervention/education plan (n = 2133)	First autism diagnosis (n = 2303)
	β (95% CI)	β (95% CI)	β (95% CI)
Age cohort			
Early childhood	Ref	Ref	Ref
Middle childhood	1.49*** (1.18, 1.81)	1.80*** (1.46, 2.15)	1.88*** (1.50, 2.27)
Adolescence	2.33*** (1.93, 2.73)	2.60*** (2.23, 2.98)	3.16*** (2.72, 3.60)
Sex			
Male	Ref	Ref	Ref
Female	0.53* (0.01, 1.05)	0.05 (− 0.34, 0.43)	0.76** (0.22, 1.31)
Race/Ethnicity			
Non-Hispanic White	Ref	Ref	Ref
Non-Hispanic Black	0.18 (− 0.45, 0.80)	− 0.28 (-0.76, 0.20)	− 0.28 (-0.82, 0.26)
Hispanic/Latinx	− 0.40* (− 0.76, − 0.04)	− 0.53** ( − 0.93, − 0.13)	− 1.00*** (− 1.49, − 0.52)
Other or Multiracial	− 0.09 (− 0.54, 0.36)	− 0.36 ( − 0.86, 0.13)	− 0.38 (− 0.83, 0.07)
Severity of ASD			
Mild	Ref	Ref	Ref
Moderate	0.10 (− 0.26, 0.46)	− 0.26 (− 0.59, 0.07)	− 0.62** (− 1.03, − 0.22)
Severe	− 0.28 (− 0.88, 0.31)	− 0.54 (− 1.11, 0.03)	− 1.35*** (− 1.92, − 0.79)
Missing	− 0.38 (− 0.80, 0.04)	0.38 (− 0.74, 1.50)	− 1.36*** (− 1.96, 0.76)
Parental education			
High school graduate (or less)	0.40 (− 0.06, 0.86)	0.59* (0.13, 1.06)	0.83** (0.29, 1.37)
AA or some college	0.26 (− 0.13, 0.65)	0.11 (− 0.26, 0.49)	0.29 (− 0.14, 0.72)
Bachelor’s Degree (or higher)	Ref	Ref	Ref
Family Income, % of FPL^a^			
< 100%	0.17 (− 0.38, 0.71)	0.28 (− 0.30, 0.87)	− 0.55 (− 1.18, 0.08)
100–199%	− 0.04 (− 0.54, 0.47)	− 0.14 (− 0.61, 0.33)	− 0.53 (− 1.14, 0.08)
200–399%	− 0.13 (− 0.52, 0.26)	− 0.04 (− 0.46, 0.39)	− 0.34 (− 0.83, 0.16)
> 400%	Ref	Ref	Ref
Family structure			
Two-parent household	Ref	Ref	Ref
Other household/missing	0.38 (− 0.02, 0.77)	0.17 (− 0.22, 0.57)	0.69** (0.25, 1.14)
Type of healthcare provider to diagnose			
Yes	Ref	Ref	Ref
No	− 0.08 (− 0.39, 0.24)	− 0.08 (− 0.40, 0.24)	− 0.10 (− 0.48, 0.29)
Type of healthcare provider to diagnose			
Specialist	Ref	Ref	Ref
Primary care physician	0.22 (− 0.23, 0.68)	0.35 (− 0.03, 0.74)	− 0.17 (− 0.66, 0.32)
School psychologist	0.77* (0.16, 1.38)	1.07*** (0.65, 1.50)	0.64* (0.05, 1.23)
Non-school psychologist	0.71** (0.17, 1.25)	1.01*** (0.52, 1.50)	1.18*** (0.59, 1.76)
Psychiatrist	1.36*** (0.67, 2.06)	1.06** (0.37, 1.74)	1.79*** (1.03, 2.54)

*p < .05; **p < .01; ***p < .001

*ASD* autism spectrum disorder. ^a^FPL = % of federal poverty level. All models were adjusted for survey year, age cohort, child and household characteristics, and the type of doctor to first diagnose the child with an ASD. Child characteristics include sex, race/ethnicity, and severity of ASD. Household characteristics parental education, % of FPL and family structure. All analyses were weighted to account for the complex survey design

### First developmental services

Compared to the Early Childhood cohort, those in the Middle Childhood cohort were almost 18 months older when they first began developmental services (β = 1.49; 95% CI, 1.18 to 1.81), and Adolescents were approximately 28 months older (β = 2.33; 95% CI, 1.93 to 2.73). When compared to non-Hispanic white children, Hispanic/Latinx children were approximately five months younger at age of first developmental services (β = 0.40, 95% CI, − 0.76 to − 0.04), the type of healthcare provider who was the first to tell the parent their child had an ASD was associated with increased age at first developmental services. Compared to kids diagnosed by specialists, children diagnosed by a counselor were approximately eight months older (school: β = 0.77; 95% CI, 0.16 to 1.38; non−school β = 0.71; 95% CI, 0.17 to 1.25) and those diagnosed by a psychiatrist were approximately 16 months older (β = 1.36; 95% CI, 0.67 to 2.06) when they first began developmental services.

### First intervention/education plan

Compared to the Early Childhood cohort, those in Middle Childhood were nearly 22 months older when they had their first plan for intervention/education (β = 1.80; 95% CI, 1.46 to 2.15), while Adolescents were more than 31 months older at their first plan (β = 2.60; 95% CI, 2.23 to 2.98). When compared to non-Hispanic white children, Hispanic/Latinx children were more than six months younger when they received their first plan (β = − 0.53; 95% CI, − 0.93 to − 0.13). Age at first plan was more than a year older for children who received their first ASD diagnosis from a psychologist (school: β = 1.07; 95% CI, 0.65 to 1.50; non-school: β = 1.01; 95% CI, 0.52 to 1.50), or a psychiatrist (β = 1.06; 95% CI, 0.37 to 1.74) compared to a specialist.

#### First ASD diagnosis

Compared to the Early Childhood cohort, those in Middle Childhood were almost 23 months older when they were first diagnosed (β = 1.88; 95% CI, 1.50 to 2.27), while Adolescents were more than three years older (β = 3.16; 95% CI, 2.72 to 3.60). When compared to non-Hispanic white children Hispanic/Latinx children were a full year younger at age of first ASD diagnosis (β = 1.00; 95% CI, −1.49 to −0.52). Children who were rated as having severe autism were diagnosed approximately 16 months earlier (β = 1.35; 95% CI, − 1.92 to − 0.79), and children with moderate autism were diagnosed about seven months earlier (β = −0.62; 95% CI, −1.03 to −0.22) compared to children with mild autism. As with the age at first plan, the type of healthcare provider that the child received their first ASD diagnosis from was also associated with increased ages at diagnosis. Compared to receiving a diagnosis from a specialist, those who were diagnosed by a psychologist were more than seven to 14 months older (school: β = 0.64; 95% CI, 0.05 to 1.23; non-school: β = 1.18; 95% CI, 0.59 to 1.76), and those who were diagnosed by a psychiatrist were more than 21 months older (β = 1.79; 95% CI, 1.03 to 2.54).

## Discussion

In this nationally representative study of children with an ASD diagnosis born between 2001–2015, the ages at which children experienced three common events in the diagnostic odyssey and the order in which the events occurred was unique to each cohort. Cohort effects were robust for all three events across all three cohorts, with a clear and consistent pattern of experiencing all three events at younger ages with each decreasing cohort. In addition, younger ages at events were observed among Hispanic/Latinx children as compared to white children, children with moderate or severe ASD symptoms as compared to children with mild symptoms, and children who received their diagnosis from a specialist as compared to children who received their diagnosis from a psychologist or psychiatrist.

When we consider the sample overall (i.e., all cohorts combined), the findings regarding the age and order of events for the overall sample were similar to the 2011 Pathways Survey data, which indicated that children began developmental services (*M* = 3.90 years, SE = 0.11[5]) before receiving an ASD diagnosis (*M* = 5.2 years, 95% CI, 4.9–5.5[6]). The ages at which they reached these events were slightly older for developmental services (*M* = 4.10 years, SE = 0.10), and slightly younger for first ASD diagnosis (*M* = 4.56 years, SE = 0.13). However, our disaggregation by cohort indicates substantial progress towards earlier ages for all three events among the youngest children in the sample.

Previous findings regarding the relationship between race/ethnicity and age of ASD diagnosis have been mixed, but a recent study on the changing demographics underlying ASD prevalence increases provides a helpful context for our findings. Nevison and Zahorodny [26] used enrollment data from the US Department of Education for 50 states to examine changes in ASD prevalence among 3–5 year-old children receiving special education or early intervention services for ASD through Part B of IDEA between 2000–2017. The authors found that the prevalence of Hispanic/Latinx children had ‘caught up’ to the prevalence of non-Hispanic white children by 2013, and that this increased identification of Hispanic/Latinx children accounts for approximately one-third of the overall increased ASD prevalence during the study period. The authors suggest that earlier age of diagnosis of Hispanic/Latinx children could be driving the increased prevalence. This demographic shift can be seen in Table 1, as the proportion of Hispanic/Latinx children increases from approximately 23% in the oldest cohort to more than 32% in the two younger cohorts. Further study is warranted to identify the specific mechanisms associated with improved identification in this population.

Children who received their diagnoses from specialists and primary care providers experienced all three events at earlier ages, which is consistent with earlier findings indicating later ages of diagnosis among those children who receive a diagnosis in the school setting [17]. This could be related to the high variability of evaluation procedures and timelines amongst state education agencies, whose primary objective in evaluating suspected cases of ASD is to determine if the autism affects the student’s educational performance [27]. Additional data regarding the identification processes in the medical and educational settings could clarify the role of the type of provider to first diagnose ASD in the diagnostic odyssey.

While these findings are encouraging in terms of improvements, it is important to consider several limitations in this study. First, this study relies on retrospective survey questions to approximate events along the pathway to diagnosis and intervention. Parents of adolescents may have a less accurate recollection of events compared to parents of much younger children. Similarly, age data were only released in years and months for children who were under age two or three years at the time of the first plan or services (respectively). Therefore, the estimates of the ages at these events will be more precise for younger children compared to the older children.

There are also several strengths of the study that are important to highlight. The large, complex survey design of the NSCH and the annual collection yielded data that are recent and nationally representative. The oversampling of younger children, children with special healthcare needs, and the ability to pool several years of data produced a large sample of children in three distinct stages of child development, thereby enabling a comparison of experiences over a seventeen-year period.

The consistent pattern of younger ages at which children with autism first receive a diagnosis, intervention/education plan, and developmental services for the youngest cohort of children is an encouraging takeaway from this study; however, more detailed data about the diagnostic odyssey would help identify how these improvements in early identification and intervention have been achieved. Future studies that collect detailed histories of events may also help clarify the role of healthcare providers along the diagnostic pathway, as well as sources of disparities.

## Conclusion

The ages at which children in the US are first diagnosed with an ASD, receive a plan for intervention or special education and begin receiving developmental services have improved over the past seventeen years. Demographic shifts in early identification have reduced disparities and highlight the need for future research to gather additional data on the diagnostic odyssey to better enable all children to achieve their full potential.

## Supplementary Information

**Additional file 1: Appendix S1. Figure S1.** Inclusion criteria and sample selection, National Survey of Children’s Health, 2016—2018 (n = 2303). **Table S1.** Adjusted linear regression associations for ages at events, National Survey of Children’s Health, 2016—2018.

## Data Availability

Data are publicly available at: https://www.census.gov/programs-surveys/nsch/data/datasets.html.

## Abbreviations

NSCH: National Survey of Children’s Health
ASD: Autism spectrum disorder
IFSP: Individualized family service plan
IEP: Individualized education plan
FPL: Federal poverty level
IDEA: Individuals with Disabilities in Education Act
