# Supplementary material for: The diagnostic odyssey of autism: a cross-sectional study of 3 age cohorts of children from the 2016–2018 National Survey of Children’s Health
Source: Child Adolesc Psychiatry Ment Health. 2021 Oct 10;15:58. doi: 10.1186/s13034-021-00409-y (PMC8504038; doi:10.1186/s13034-021-00409-y)
Supplement: Supplementary file 1 — Additional file 1: Appendix S1. Figure S1. Inclusion criteria and sample selection, National Survey of Children’s Health, 2016—2018 (n = 2303). Table S1. Adjusted linear regression associations for ages at events, National Survey of Children’s Health, 2016—2018. [file 13034_2021_409_MOESM1_ESM.docx]

***Additional file 1: Appendix S1. Figure S1***

**Inclusion criteria and sample selection,** **National Survey of Children’s Health, 2016—2018 (n=2,303)**

2016

N = 50,212

2017

N = 21,599

2018

N = 30,530

2016-18

N = 102,341

2016-18

N = 102,341

Age 2–17 years old

N = 94,303

N = 2,688

Ever told by a doctor or health care provider that this child has autism, autism spectrum disorder (ASD), Asperger’s disorder or pervasive developmental delay (PDD) (i.e., an ASD)

Ever had a plan for early intervention or special education **OR** ever received special services to meet developmental needs such as speech, occupational therapy, etc.

N = 2,303

N = 2,133

N = 2,164

Age 2–17 AND ever diagnosed with an ASD AND ever had a special education or early intervention plan (e.g., Individualized Family Service Plan or Individualized Education Plan)

Age 2–17 AND ever been diagnosed with an ASD AND received special services to meet his or her developmental needs such as speech, occupational or behavioral therapy

***Additional file 1: Appendix S2. Table S1***

**Adjusted linear regression associations for ages at events, National Survey of Children’s Health, 2016—2018**

|  | **First Developmental Services**  (n=2,164) | | **First Intervention/Education Plan**  (n=2,133) | | **First Autism Diagnosis**  **(**n=2,303) | |
| --- | --- | --- | --- | --- | --- | --- |
|  | Model 1  𝜷 (95% CI) | Model 2  𝜷 (95% CI) | Model 1  𝜷 (95% CI) | Model 2  𝜷 (95% CI) | Model 1  𝜷 (95% CI) | Model 2  𝜷 (95% CI) |
| **Age Cohort** | | | | | | |
| Early Childhood | Ref. | - | Ref. | - | Ref. | - |
| Middle Childhood | 1.51*** **(1.19, 1.82)** | - | 1.90*** **(1.52, 2.29)** | - | 1.88*** **(1.50, 2.26)** | - |
| Adolescence | 2.33*** **(1.97, 2.70)** | - | 2.65*** **(2.31, 3.00)** | - | 3.26*** **(2.80, 3.73)** | - |
| **Sex** | | | | | | |
| Male | Ref. | Ref. | Ref. | Ref. | Ref. | Ref. |
| Female | 0.53 (-0.08, 1.14) | 0.59* **(0.03, 1.16)** | -0.02 (-0.49, 0.45) | 0.07 (-0.38, 0.52) | 0.87* **(0.15, 1.60)** | 0.95** **(0.30,1.59)** |
| **Race/Ethnicity** | | | | | | |
| Non-Hispanic White | Ref. | Ref. | Ref. | Ref. | Ref. | Ref. |
| Non-Hispanic Black | 0.10  (-0.66, 0.86) | 0.28  (-0.42, 0.98) | -0.55*  **(-1.09, -0.02)** | -0.28  (-0.75, 0.18) | -0.70*  **(-1.33, -0.08)** | -0.42  (-0.96, 0.12) |
| Hispanic/Latinx | -0.51*  **(-0.92, -0.09)** | -0.32  (-0.72, 0.08) | -0.55  (-1.13, 0.03) | -0.35  (-0.89, 0.19) | -1.44*** **(-1.97, -0.92)** | -1.15***  **(-1.70, -0.60)** |
| Other or Multiracial | -0.34  (-0.83, 0.15) | -0.15  (-0.63, 0.32) | -0.66*  **(-1.17, -0.15)** | -0.46  (-0.96, 0.04) | -0.80**  **(-1.36, -0.23)** | -0.50  (-1.03, 0.02) |
| **Severity of ASD**^a^ | | | | | | |
| Mild | Ref. | Ref. | Ref. | Ref. | Ref. | Ref. |
| Moderate | 0.13  (-0.31, 0.56) | 0.03  (-0.38, 0.44) | -0.19  (-0.58, 0.19) | -0.28  (-0.63, 0.08) | -0.75**  **(-1.27, -0.24**) | -0.84***  **(-1.33, -0.36)** |
| Severe | -0.23  (-0.78, 0.32) | -0.26  (-0.77, 0.26) | -0.59  (-1.20, 0.01) | -0.63*  **(-1.18, -0.07)** | -1.40***  **(-2.03, -0.78)** | -1.39***  **(-1.93, -0.85)** |
| Missing | -0.45  (-1.05, 0.15) | -0.56*  **(-1.05, -0.07)** | 0.62  (-0.61, 1.85) | 0.48  (-0.78, 1.75) | -1.50**  **(-2.54, -0.45)** | -1.61***  **(-2.46, -0.77)** |
| **Parental Education** | | | | | | |
| High School Graduate (or less) | 0.23  (-0.27, 0.73) | 0.36  (-0.11, 0.84) | 0.45  (-0.08, 0.99) | 0.60*  **(0.10, 1.09)** | 0.03  (-0.59, 0.66) | 0.21  (-0.35, 0.78) |
| AA or Some College | 0.23  (-0.17, 0.64) | 0.23  (-0.17, 0.63) | 0.11  (-0.29, 0.52) | 0.09  (-0.29, 0.48) | -0.14  ( -0.65, 0.38) | -0.15  (-0.73, 0.43) |
| Bachelor’s Degree  (or more) | Ref. | Ref. | Ref. | Ref. | Ref. | Ref. |
| **Family Income, % of FPL**^b^ | | | | | | |
| <100% | 0.21  ( -0.39, 0.80) | 0.31  (-0.28, 0.90) | 0.25  (-0.37, 0.87) | 0.39  (-0.25, 1.02) | 0.21  (-0.39, 0.80) | 0.39  ( -0.25, 1.02) |
| 100% – 199% | 0.08  (-0.42, 0.58) | 0.23  (-0.25, 0.71) | -0.12  (-0.60, 0.35) | 0.03  (-0.40, 0.45) | 0.08  (-0.42, 0.58) | 0.03  (-0.40, 0.45) |
| 200% – 399% | -0.07  ( -0.51, 0.38) | 0.00  (-0.42, 0.42) | -0.00  (-0.47, 0.46) | 0.04  (-0.40, 0.48) | -0.07  (-0.51, 0.38) | 0.04  (-0.40, 0.48) |
| > 400% | Ref. | Ref. | Ref. | Ref. | Ref. | Ref. |
| **Family Structure** | | | | | | |
| Two-Parent Household | Ref. | Ref. | Ref. | Ref. | Ref. | Ref. |
| Other Household/  Missing | 0.53*  **(0.04, 1.03)** | 0.52*  **(0.08, 0.95)** | 0.26  (-0.17, 0.69) | 0.25  (-0.14, 0.64) | 0.69*  **(0.16, 1.22)** | 0.64**  **(0.17, 1.11)** |
| **Presence of Older Sibling** | | | | | | |
| Yes | Ref. | Ref. | Ref. | Ref. | Ref. | Ref. |
| No | 0.50*  **(0.12, 0.87)** | 0.12  (-0.24, 0.49) | 0.35  (-0.15, 0.86) | -0.03  (-0.48, 0.41) | 0.84**  **(0.34, 1.34)** | 0.28  (-0.19, 0.74) |
| **Type of Healthcare Provider to Diagnose** | | | | | | |
| Specialist | Ref. | Ref. | Ref. | Ref. | Ref. | Ref. |
| Primary Care Physician | 0.23  (-0.27, 0.74) | 0.26  (-0.19, 0.72) | 0.40  (-0.03, 0.82) | 0.46*  **(0.09, 0.84)** | -0.17  (-0.72, 0.38) | -0.10  ( -0.61, 0.40) |
| School Psychologist | 0.94**  **(0.30, 1.58)** | 0.88**  **(0.31, 1.46)** | 1.16***  **(0.69, 1.63)** | 1.14***  **(0.74, 1.53)** | 0.85*  **(0.08, 1.63)** | 0.82*  **(0.16, 1.48)** |
| Non-School Psychologist | 0.63  (-0.01, 1.28) | 0.64*  (0.05, 1.22) | 1.01**  **(0.37, 1.66)** | 1.03***  **(0.51, 1.55)** | 1.31***  **(0.58, 2.04)** | 1.28***  **(0.67, 1.89)** |
| Psychiatrist | 1.65***  **(0.89, 2.40)** | 1.37***  **(0.63, 2.10)** | 1.35***  **(0.67, 2.03)** | 1.08**  **(0.43, 1.73)** | 2.25***  **(1.23, 3.26)** | 1.89***  **(0.96, 2.82)** |
| * *p*<.05; ** *p*<.01; *** *p*<.001  ^a^ASD= autism spectrum disorder. ^b^FPL= % of federal poverty level.  Model 1 was adjusted for survey year. Beta estimates for all covariates in Model 2 were produced in separate models, adjusted for survey year and age cohort. All analyses were weighted to account for the complex survey design. | | | | | | |
